# GATA-*3* Suppression by DNAzyme Modulates Interleukin-10 and Liver Injury Markers in *db*/*db* Mice

**DOI:** 10.3390/biology15010089

**Published:** 2025-12-31

**Authors:** Layla Al-Mansoori, Asma A. Elashi, Laila Hedaya, Maha Alser, Shamma Almuraikhy, Najeha Anwardeen, Hend Al-Jaber, Suhad Hussain, Hamda A. Al-Naemi, Vijay Govindharajan, Rafif Mahmood Al-Saady, Mohammed Imad Malki, Khaled Naja, Mohamed A. Elrayess

**Affiliations:** 1 Biomedical Research Center, QU Health, Qatar University, Doha P.O. Box 2713, Qatar; almansouri@qu.edu.qa (L.A.-M.); asma.elashi@qu.edu.qa (A.A.E.); laila.hedaya@qu.edu.qa (L.H.); maha.alser@qu.edu.qa (M.A.); salmuraikhy@qu.edu.qa (S.A.); n.anwardeen@qu.edu.qa (N.A.); haljaber@qu.edu.qa (H.A.-J.); suhadhussain@outlook.com (S.H.); khaled.naja@qu.edu.qa (K.N.); 2 Laboratory Animal Research Centre, Qatar University, Doha P.O. Box 2713, Qatar; halnaemi@qu.edu.qa (H.A.A.-N.); vijay.kanth@qu.edu.qa (V.G.); 3 Department of Biological and Environmental Sciences, Qatar University, Doha P.O. Box 2713, Qatar; 4 College of Medicine, QU Health, Qatar University, Doha P.O. Box 2713, Qatar; rafif@qu.edu.qa (R.M.A.-S.); momalki@qu.edu.qa (M.I.M.)

**Keywords:** obesity, type 2 diabetes, GATA-3, *db*/*db* mice, adipogenesis

## Abstract

Obesity plays a crucial role in the progression of insulin resistance and type 2 diabetes which are related to inflammation and liver disease. GATA-3 is a transcription factor that is involved in adipogenesis and inflammation. Therefore, it could be a potential therapeutic target for obesity-associated metabolic disorders. This study aimed to examine the effects of GATA-3 suppression on body weight, fat depot redistribution, liver histopathology, and inflammatory markers in transgenic *db*/*db* obese mice. Male *db*/*db* mice received subcutaneous injections of GATA-3-specific DNAzyme (hgd40; 10 or 100 µg/mL), pioglitazone (as a positive control), or vehicle only (as a negative control), twice weekly for two weeks. Body weight, organ weights, liver histopathology, mRNA expression of selected genes and serum cytokine levels were assessed. *GATA-3* expression was not region specific, and its suppression did not significantly affect fat depot distribution or organ weights. However, the low dose of hgd40 accelerated body weight gain transiently. It also increased *Il10* mRNA expression in the liver and significantly increased IL-10 protein concentration in the serum. In addition, a high dose of hgd40 resulted in a marked decrease in hepatocyte ballooning degeneration. These findings suggest that GATA-3 suppression may modulate inflammation and liver injury in obesity, warranting further investigation into its therapeutic potential for obesity-related metabolic disorders.

## Simple Summary

Obesity often leads to liver problems and inflammation, which can worsen conditions like type 2 diabetes. This study explores whether reducing the activity of an intracellular protein called GATA-3, which is involved in fat cell development and inflammation, might offer benefits. We treated obese diabetic mice with a special enzyme-based treatment that targets GATA-3. We found that the treatment did not change how fat was distributed in the body, but it did reduce signs of liver damage and increase levels of an anti-inflammatory protein called interleukin-10. Our results suggest that targeting GATA-3 could be a promising way to protect the liver and reduce inflammation in obesity-related diseases, potentially leading to new treatments for conditions like fatty liver disease and diabetes.

## 1. Introduction

Obesity plays a crucial role in the progression of insulin resistance (IR), type 2 diabetes (T2D), and fatty liver disease [1,2]. These metabolic disorders are associated with impaired adipogenesis and persistent low-level inflammation [2,3,4]. Dysregulation of adipogenesis affects the systemic metabolism of adipose tissues through several mechanisms, including adipocyte hypertrophy (enlargement of adipose tissues) and adipose tissue inflammation [5]. This leads to the disruption and remodeling of adipose tissues, increased local inflammation and the formation of ectopic fat deposits, which eventually contribute to the progression of IR and fatty liver disease [2,6].

Obesity-induced adipose tissue inflammation is characterized by the secretion of pro-inflammatory cytokines and adipokines including interleukin-1 beta (IL-1β), interleukin-6 (IL-6), and tumor necrosis factor alpha (TNF-α), in addition to chemokines, such as monocyte chemoattractant protein-1 (MCP-1) [7,8]. The secretion of these immune mediators results in the inhibition of insulin receptor substrate-1 (IRS-1) expression and glucose transporter type 4 (GLUT4) translocation [9,10,11,12], both of which are involved in insulin signaling and glucose uptake. TNF-α also increases the formation of reactive oxygen species and activates several pathways which cause apoptosis and hepatic injury, aggravating liver disease [13]. Furthermore, impaired adipogenesis involves the alteration of key transcription factors that regulate preadipocyte–adipocyte transition, such as peroxisome proliferator-activated receptor gamma (PPARγ) and CCAAT/enhancer-binding protein alpha (C/EBPα) [14]. Together, these mechanisms adversely affect insulin sensitivity during adipogenesis and lipid metabolism-related genes, contributing significantly to the progression of obesity-related IR and liver steatosis [15,16].

GATA-binding protein 3 (GATA-3) is a key transcription factor that regulates various biological processes, including immune function and adipogenesis. In particular, it is essential for the differentiation of T helper 2 (Th2) cells, in which it stimulates the release of cytokines, including IL-6 [17] and interleukin-4 (IL-4) [18]. Thus, GATA-3 was proposed as a therapeutic target for diseases mediated by T cells, such as asthma and colitis [19,20]. Specifically, an emerging class of nucleic acid therapeutics, called DNAzymes, was employed for this purpose. A DNAzyme primarily consists of a DNA-based catalytic loop that cleaves RNA molecules at a specific target determined by two flanking oligonucleotide arms that are designed to bind the desired RNA sequence [21]. Intranasal administration of hgd40, a DNAzyme that specifically binds and cleaves *GATA-3* mRNA, has been shown to be a promising treatment strategy for chronic asthma in preclinical studies, while having a favorable toxicity profile [22,23]. Additionally, rectal delivery of hgd40 was demonstrated to be effective for the prevention and treatment of colitis in mice [20].

Studies have also linked GATA-3to adipogenesis and tissue inflammation. Preadipocytes express higher levels of GATA-3 in comparison to differentiated adipocytes [24]. Further investigation revealed that GATA-3 regulates the expression of PPARγ and C/EBP, suppressing the differentiation of preadipocytes into adipocytes [25,26]. In addition, GATA-3 was shown to regulate microRNA-370/high mobility group box 1 (miR-370/HMGB1) signaling pathway, promoting autophagy and hepatic stellate cell activation, which contributes to liver fibrosis [27]. Collectively, these findings highlight GATA-3 as a potential candidate target for modulating adipogenesis and inflammation, improving IR and liver injury.

We have previously reported that *GATA-3* mRNA is highly expressed in the stromal vascular fraction-derived preadipocytes and transitioned adipocytes of IR obese individuals, compared to insulin-sensitive (IS) counterparts [28,29]. Interestingly, for both IR and IS obese individuals, *GATA-3* expression is higher in omental adipose tissue (OMAT) than subcutaneous adipose tissue (SAT) [28]. The inhibition of GATA-3 utilizing hgd40 in mouse preadipocytes [28] and primary human preadipocytes [30] resulted in enhancing adipogenesis by increasing adipogenic gene expression of PPARγ [28,31]. It also improved insulin signaling through the downregulation of p70 ribosomal protein S6 kinase (p70S6K) [31]. Further, in a proof-of-concept study, we demonstrated that the subcutaneous injection of hgd40 in the right flank region of normal-weight male *BALB/c* mice (lean) significantly increased the adipose tissue weight in the injection site compared to the untreated site. It also resulted in lower weight compared to that of vehicle-treated control mice [31]. The observed results may be explained by the mobilization of omental fat, triggered by expanded subcutaneous storage space through GATA-3 inhibition, which improved the overall insulin sensitivity in the treated animals.

In this study, we aim to examine the effects of hgd40 on body weight, liver histopathology, fat redistribution, and inflammatory markers. Transgenic *db*/*db* mice models were chosen as they demonstrate key characteristics of obesity and T2D [32]. They were selected to be between 12 and 16 weeks old, a time by which *db*/*db* mice are at near maximal adiposity [33,34,35,36,37], to determine whether GATA-3 suppression can redistribute fat depots. Pioglitazone was utilized as a positive control due to its previously demonstrated ability to decrease visceral fat accumulation and increase the formation of new adipocytes in the subcutaneous femoral adipose tissue of obese individuals [38]. The weights of the pancreas, liver, skeletal muscles, and brain were assessed as these organs play important roles in the regulation of insulin sensitivity and glucose homeostasis and their function can be affected by obesity and fat accumulation [39,40].

## 2. Materials and Methods

### 2.1. Liposome Preparation

Liposomes carrying the control (scrambled DNA) or treatment (hgd40 at 10 µg/mL, or 100 µg/mL) were prepared by ethanol-based pro-liposome technology, following previously published methods [41,42]. The scrambled DNA and hgd40 were obtained from Integrated DNA Technologies IDT (Coralville, IA, USA). Briefly, 50 mg of phospholipid, Sigma-Aldrich (St. Louis, MO, USA), was mixed with 100 mL of absolute ethanol, and the mix was allowed to dissolve at 70 °C. To this solution, 50 mg of cholesterol was added, and the mixture was further incubated in a 70 °C water bath to dissolve. Subsequently, treatments were added to their corresponding mixes to reach the following concentrations: 100 µg/mL of scrambled DNA (vehicle control treatment), 1 µM pioglitazone (cat. no. 112529-15-4), Sigma-Aldrich (USA), as positive control [31], 10 µg/mL of hgd40 (GATA-3 DNAzyme 10 µg) and 100 µg/mL of hgd40 (GATA-3 DNAzyme 100 µg). Then, they were mixed vigorously for 4 min. This was followed by incubation at room temperature for 2 h, and then sonication for 10 min. To remove any residual titanium particles from the sonicator probe, the liposomes were centrifuged at 12,000 rpm for 15 min (Centurion Scientific, Chichester, UK), followed by collecting the supernatant and filtration through a 0.2 µm filter (sterilization) prior to injection into animals. The chosen high dose of 100 µg/mL matches the concentration validated in our previous study [31] where subcutaneous hgd40 administration effectively suppressed GATA-3, enhanced adipogenesis, and improved insulin sensitivity in lean mice without toxicity. The 10 µg/mL low dose was included as a one-log step-down to probe dose–response effects while staying compatible with the ethanol-based pro-liposome formulation.

### 2.2. In Vivo Experimental Design and Treatment

Adult male (12–16 weeks old) leptin receptor-deficient *db*/*db* transgenic mice were obtained from The Jackson Laboratory (Bar Harbor, ME, USA) and maintained by the Laboratory Animal Research Center (LARC) at Qatar University (QU). Animals were housed in individually ventilated cages (IVC) under standard conditions: room temperature of 18–22 °C, relative humidity of 30–70% and 12/12 h light/dark cycle. The mice were fed with normal chow diet and drinking water ad libitum. All animal procedures were performed according to approved institutional ethical rules and regulations and were approved by Qatar University—Institutional Animal Care and Use Committee (QU-IACUC 024/2020).

A total of 49 animals were used in this study and randomly assigned into four experimental groups: (A) Vehicle control group, treated with 100 μL of DNAzyme-free liposomes (*n* = 10); (B) Positive control group, treated with 1 μM pioglitazone loaded-liposomes (40 mg/Kg; *n* = 13); (C) GATA-3 DNAzyme (10 µg) group, treated with 100 μL of 10 µg/mL hgd40 loaded-liposomes (*n* = 13), and (D) GATA-3 DNAzyme (100 µg) group, treated with 100 μL of 100 µg/mL hgd40 loaded-liposomes (*n* = 13). Treatments were administered by subcutaneous injections to the right flank region, twice a week, for 2 weeks (total 4 injections per animal). Animal body weights were recorded before, 1 week after, and 2 weeks after the intervention course. All the experimental animals were humanely euthanized by CO_2_ inhalation followed by physical secondary method as per AVMA Guidelines for the Euthanasia of Animals (2020).

Organs of interest (right subcutaneous, left subcutaneous, omental, inguinal, and brown adipose tissues, as well as pancreas, skeletal muscle, brain, and liver tissues) were collected and weighed. The collection of different adipose tissues and their anatomical boundaries was based on [43,44]. Right and left subcutaneous adipose tissues (SATs) were collected from the lateral abdominal wall (dorso lumbar region). Omental adipose tissue (OMAT) was collected from the greater omentum lined across the surface of the stomach. Inguinal adipose tissue was collected from the inner thigh region. Brown adipose tissue was collected from the interscapular region. Skeletal muscles were collected from the hind limb region, around the femur (from proximal to distal region). Reproducibility and consistency were ensured throughout the organ collection procedures. The sample size was standardized by collecting the entire organ sample from each animal. All the dissections were performed by the same investigator. After weighing, liver samples were fixed in 10% formalin, followed by histological examination. Other liver samples were snap frozen in dry ice and stored at −80 °C for further molecular analyses.

### 2.3. Histopathology Assessment

Further histological processing of the liver tissue samples was carried out by Al Borg Diagnostics (Doha, Qatar) according to the general Hematoxylin and Eosin (H&E) histopathology protocol. In brief, the tissues were serially dehydrated in ethanol (70–100%, 1 h per concentration), cleared twice with xylene, and paraffin-processed. Then, the tissues were sectioned into 5 μm thick sections, stained following the common H&E procedure, mounted with DPX mounting media, and covered with a cover slip. The slides were then kept on a slide warmer for 16–24 h. The sections were examined and imaged under a light microscope. Histopathological analysis was conducted by two independent histopathologists to assess the percentages of normal, ballooning, and fatty liver cells. All fields were evaluated, and all cells were counted. The slides were screened at 10×, and then confirmed at 20× and 40×.

### 2.4. Gene Expression Assessment

Gene expression was assessed in liver and SAT samples from the four experimental groups by the RT-PCR method. From each liver sample, 20 mg of frozen (−80 °C) tissue was cut and physically homogenized with a tissue grinder and a pestle. RNA was extracted using Qiazol lysis reagent (Qiagen, Hilden, Germany, cat. no. 79306) following the TRIzol general method provided by the manufacturer. Then, 1700 ng of total RNA was used for first-strand cDNA synthesis using High-capacity cDNA synthesis kit (Applied Biosystems, Foster City, CA, USA, cat. no. 4368814) following the manufacturer’s protocol. After that, 170 ng of the cDNA product was used as a template for RT-PCR using Luna^®^ Universal qPCR Master Mix (New England Biolabs, Ipswich, MA, USA, cat. no. M3003E) following the manufacturer’s instructions. From each SAT sample, 50–70 mg of frozen tissue was physically homogenized in TRIzol reagent (Invitrogen, Carlsbad, CA, USA, cat. no. 15596026) with a tissue grinder and a pestle. The homogenized samples were centrifuged at 12,000× *g* for 10 min at 4 °C, and the clear supernatant containing the RNA was separated from the insoluble material and the top fatty layer. Then, RNA was extracted following the manufacturer’s protocol. A total of 150 ng RNA was used to synthesize cDNA using High-capacity cDNA synthesis kit (Applied Biosystems, cat. no. 4368814) following the manufacturer’s protocol. 1 µL of the cDNA product was used as a template for RT-PCR using Luna^®^ Universal qPCR Master Mix (New England Biolabs, cat. no. M3003E) following the manufacturer’s instructions.

The primers listed in Table 1 were used for assessing the expression of genes of interest. For all samples, RT-PCR was run using the 7500 RT-PCR System from Applied Biosystems following PCR conditions recommended by the kit. *Gapdh* was used as a housekeeping gene for normalization. Data analysis was performed using the ∆∆Ct-based method.

### 2.5. Serum Cytokines Assay

Serum samples collected from the treated mice were analyzed for a panel of selected cytokines using Mouse ProcartaPlex Mix&Match 6-plex kit (#PPX-06-MXH6CWJ, Invitrogen, Waltham, MA, USA). The assay was performed following the manufacturer’s instructions. In brief, serum samples were incubated with antibody-conjugated magnetic beads, followed by washing and incubation with biotinylated detection antibodies. Then, excess antibodies were washed out, and the beads were incubated with streptavidin-phycoerythrin fluorescent reporter. A final washing step was performed before resuspending the beads in reading buffer. LABScan3D™ machine (One Lambda, Thermo Fisher Scientific, Waltham, MA, USA) and Luminex xPONENT^®^ for FLEXMAP 3D Software (version 4.3, Luminex Corp., Austin, TX, USA) were used to measure the fluorescent signals and acquire data.

### 2.6. Statistical Analysis

Statistical analyses were performed using GraphPad Prism software (version 10.6.0, GraphPad Software, Boston, MA, USA). Data distribution was assessed using the Shapiro–Wilk normality test. Homogeneity of variance was evaluated with the Brown-Forsythe test. Comparisons between the right and left flank regions of the SAT were conducted using a paired *t*-test after ensuring normality. The Wilcoxon signed-rank test was used for paired data that are not normally distributed. To assess the effect of GATA-3-specific DNAzyme on total body weight over time, repeated measures one-way ANOVA was used for parametric data. The Friedman test was used for non-parametric data. Unpaired data with >2 groups were analyzed using one-way ANOVA followed by Dunnett’s post hoc test, after ensuring normality and homogeneity of variance. Otherwise, the Kruskal–Wallis test followed by Dunn’s multiple comparisons test was used for non-normally distributed data. Only adjusted *p*-values were reported for multiple comparisons. A *p*-value (or adjusted *p*-value, when applicable) of <0.05 was considered statistically significant.

## 3. Results

### 3.1. GATA-3 Expression Does Not Differ Between the Treated and Untreated Sides of the SAT upon GATA-3 DNAzyme Treatment

**Figure 1:**
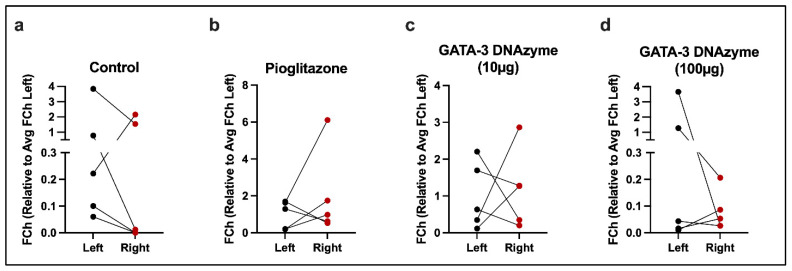
*GATA-3* mRNA expression of the left and right subcutaneous adipose tissues. Reverse transcriptase PCR (RT-PCR) was used to examine *GATA-3* mRNA expression in the subcutaneous adipose tissues (SATs) from the left (untreated side) and right (treated side) flank regions of *db*/*db* mice (*n* = 5/group) on day 14 of treatment. Each pair of connected dots represents data from a single mouse at the indicated SAT regions. *Gapdh* was used as an internal control. Results are shown as fold change (FCh) relative to the average FCh (Avg FCh) of *GATA-3* mRNA expression in the left SAT. (**a**) Control: mice treated with DNAzyme-free liposomes. (**b**) Pioglitazone: mice treated with liposomes loaded with 1 µM of pioglitazone (40 mg/Kg). (**c**,**d**) GATA-3 DNAzyme: mice treated with liposomes loaded with GATA-3 specific DNAzyme (hgd40) at (**c**) (10 µg/mL) or (**d**) (100 µg/mL). For statistical analysis of (**a**–**c**), paired *t*-test was performed after confirming normality. For statistical analysis of (**d**) Wilcoxon matched pairs signed rank test was used due to violation of normality. A *p*-value of <0.05 was considered statistically significant. Due to the lack of significant/marginally significant changes in (**a**–**d**), no *p*-values are shown.

We have previously shown that *GATA-3*-specific DNAzyme (hgd40) significantly suppressed *GATA-3* expression in 3T3L-1 mouse preadipocytes [28]. To examine whether subcutaneous hgd40 injections of *db*/*db* mice cause region-specific GATA-3 suppression, *GATA-3* mRNA expression was compared in the left (untreated) versus right (treated) SATs at the end of the treatment period. No significant difference was identified in any of the experimental groups (Figure 1a–d).

### 3.2. Low Dose GATA-3 DNAzyme Transiently Accelerates Total Body Weight Gain of db/db Mice

**Figure 2:**
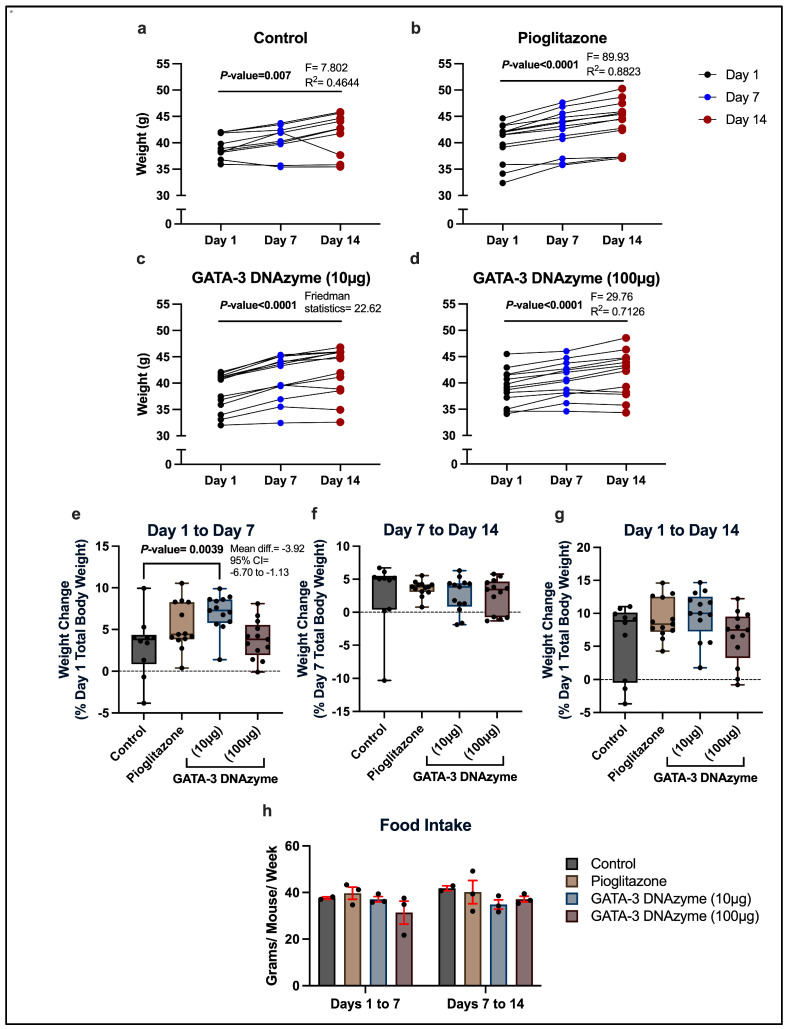
The effect of GATA-3 DNAzyme treatment on total body weight of *db*/*db* mice. (**a**–**d**) Each triplet of connected dots represents the total body weight measurements (in grams) of a single mouse at the indicated experimental days. (**a**) Control: mice treated with DNAzyme-free liposomes (*n* = 10). (**b**) Pioglitazone: mice treated with liposomes loaded with 1 µM of pioglitazone (40 mg/Kg) (*n* = 13). (**c**,**d**) GATA-3 DNAzyme: mice treated with liposomes loaded with GATA-3-specific DNAzyme (hgd40) at (**c**) (10 µg/mL; *n* = 13) or (**d**) (100 µg/mL; *n* = 13). (**e**–**g**) The rate of weight change in the indicated experimental groups, expressed as the percentage of weight difference relative to the initial total weight at the indicated timepoints. Each dot represents data from a single mouse. For the control, *n* = 10; for each of the other experimental groups, *n* = 13. Results are presented as box and whisker plots where whiskers illustrate the minimum and maximum values. (**h**) Food intake of group-housed mice at the indicated time intervals. Each dot represents the average food intake per mouse calculated for a single cage (2 cages for the control and 3 cages for each of the other experimental groups). Error bars are expressed as mean ± standard error of mean (SEM). For statistical analysis of (**a**,**b**,**d**), repeated measures one-way ANOVA was performed with Geisser-Greenhouse correction, after confirming normality. For (**c**), non-parametric Friedman test was used. For (**e**,**h**), one-way ANOVA was used after confirming normality and homogeneity of variance, followed by Dunnett’s multiple comparisons test. For (**f**,**g**), Kruskal–Wallis’s test was used due to non-normal distribution, followed by Dunn’s multiple comparisons test. All the experimental groups were compared to the control group in (**e**–**h**). A *p*-value of <0.05 was considered statistically significant. *p*-values were only indicated for significant or marginally significant results.

The average total body weight of the treated mice has significantly increased over time in all the groups, regardless of the treatment type (Figure 2a–d; *p* ≤ 0.007). Despite the overall trend of weight increase during the experimental period, some mice gained little to no weight, or even lost weight from day 1 to day 7 and/or from day 7 to day 14 (Figure 2a–d).

Examining inter-group differences in the rate of weight change over the experimental period revealed that mice treated with 10 µg/mL hgd40 exhibited a significantly greater increase in body weight from day 1 to day 7 compared to the control group (Figure 2e). Although there was no significant difference between these groups in the rate of weight change from day 7 to day 14, the control group tended to gain weight at a slightly higher rate during this period (Figure 2f). This has compensated for the initially observed difference, leading to an insignificant inter-group difference in the net weight change from day 1 to day 14 (Figure 2g).

The average initial total body weight of the mice treated with 10 µg/mL hgd40 was not significantly different from the control group (Dunn’s multiple comparisons test: control vs. GATA-3 DNAzyme (10 µg/mL), adjusted *p*-value > 0.9999), eliminating the possibility that the increased rate of weight gain is influenced by the initial total body weight. In addition, the average amount of food consumed per mouse in each week was similar between all the groups, excluding food intake as an explanation for this observation (Figure 2h).

### 3.3. GATA-3 DNAzyme Treatment Does Not Affect Body Fat Distribution in db/db Mice

**Figure 3:**
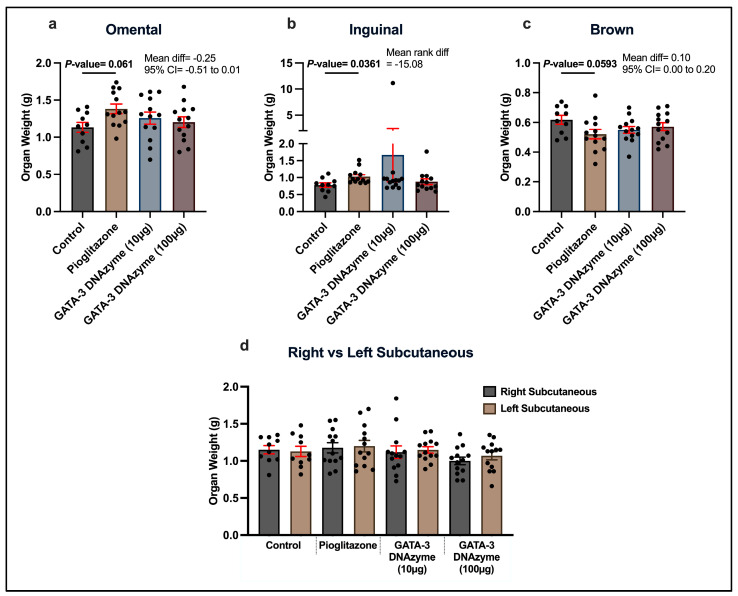
The effect of GATA-3 DNAzyme treatment on different fat depots of *db*/*db* mice. Weights in grams of (**a**) omental adipose tissue, (**b**) inguinal adipose tissue, (**c**) brown adipose tissue, and (**d**) subcutaneous adipose tissue of the right and left flank regions, on day 14 of treatment. Each dot represents data from a single mouse. For the control, *n* = 10; for each of the other experimental groups, *n* = 13. Statistical analyses of (**a**,**c**) were performed using one-way ANOVA, followed by Dunnett’s multiple comparisons test after ensuring normality and homogeneity of variance. Statistical analysis of (**b**) was performed using Kruskal–Wallis’s test, followed by Dunn’s multiple comparisons test due to violation of normality. For (**d**), two-tailed paired *t*-test was used for normally distributed groups and Wilcoxon signed-rank test was used for non-normally distributed groups. All the experimental groups were compared to the control group in (**a**–**c**). The left side was compared to the right side in (**d**). Error bars are expressed as mean ± SEM. A *p*-value of <0.05 was considered statistically significant. *p*-values were only indicated for significant or marginally significant results.

Treating *db*/*db* mice with either 10 µg/mL or 100 µg/mL hgd40 did not cause significant changes in the weights of omental and inguinal fat depots. In contrast, pioglitazone caused a trend of increase in the OMAT weight (*p* = 0.061) and a significant increase in the inguinal adipose tissue weight in comparison to the negative control (Figure 3a,b). Additionally, hgd40 had no significant effect on the brown adipose tissue (BAT), as opposed to pioglitazone which resulted in a marginally significant (*p* = 0.059) decrease in the BAT weight compared to the control (Figure 3c). No significant weight difference was observed between the SATs at the injection site (right side) and the untreated site (left side) in *db*/*db* mice after pioglitazone or hgd40 treatments (Figure 3d).

### 3.4. GATA-3 DNAzyme Treatment Does Not Change Organ Weights of Pancreas, Liver, Muscles and Brain of db/db Mice

**Figure 4:**
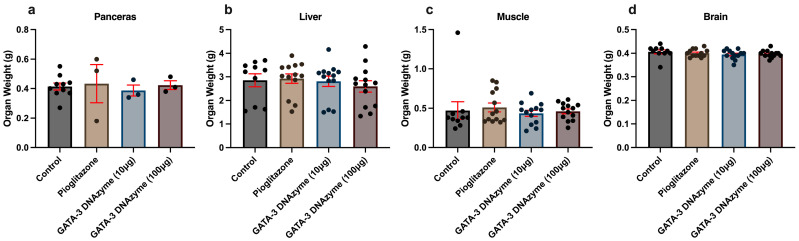
The effect of GATA-3 DNAzyme treatment on the weights of different organs. Weight in grams (g) of (**a**) pancreas, (**b**) liver, (**c**) skeletal muscles, and (**d**) brain of *db*/*db* mice from the indicated treatment groups. Each dot represents data from a single mouse. For the control, *n* = 10; for each of the other experimental groups, *n* = 13 except in (**a**) where *n* = 3. Statistical analysis of (**a**–**d**) was performed using Kruskal–Wallis’s test, followed by Dunn’s multiple comparisons test due to violation of normality. All the experimental groups were compared to the control group. Results are expressed as mean ± SEM. A *p*-value of <0.05 was considered statistically significant. Due to the lack of significant/marginally significant changes in (**a**–**d**), no *p*-values are shown.

To investigate whether GATA-3 suppression exerts systemic effects, the weights of the pancreas, liver, skeletal muscles, and brain were assessed. Neither pioglitazone nor hgd40 had a significant effect on the weight of any of these organs (Figure 4a–d).

### 3.5. GATA-3 DNAzyme Decreases Ballooning Degeneration in the Liver of db/db Mice

**Figure 5:**
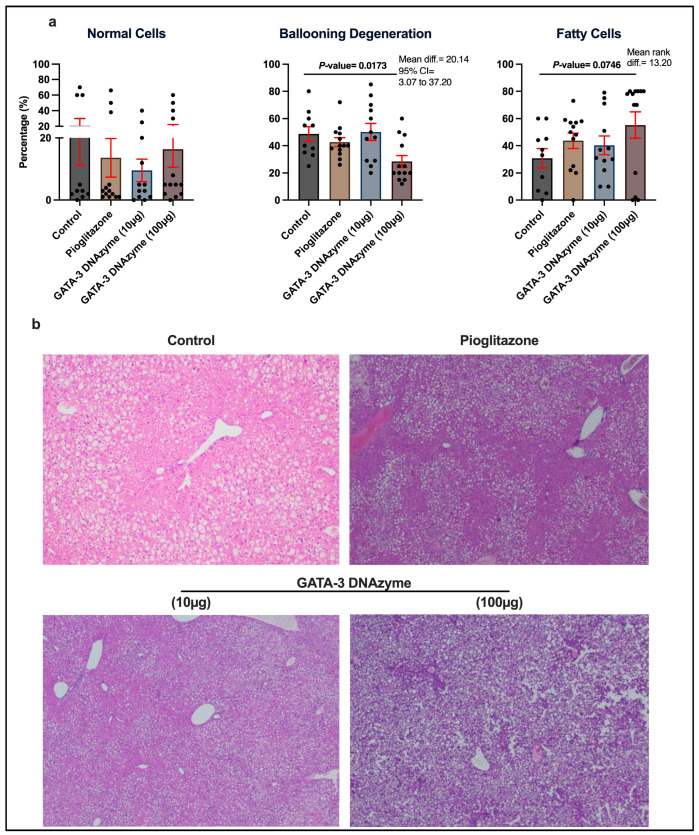
Liver histopathological alterations induced by GATA-3 DNAzyme treatment in *db*/*db* mice. (**a**) The proportions of hepatocytes with no pathological changes (normal cells), hepatocytes exhibiting ballooning degeneration (ballooning degeneration), and hepatocytes with fatty changes (fatty cells) in paraffin-embedded liver cross-sections from treated mice. Each dot represents the percentage of the specified cell type out of the total hepatocytes within an entire cross-section from a single mouse. For the control, *n* = 10; for each of the other experimental groups, *n* = 13. Statistical analyses of (**a**) normal cells and fatty cells were performed using Kruskal–Wallis’s test, followed by Dunn’s multiple comparisons test due to violation of normality. Statistical analysis of (**a**) ballooning degeneration was performed using one-way ANOVA, followed by Dunnett’s multiple comparisons test after ensuring normality and homogeneity of variance. All experimental groups were compared to the control group. Results are expressed as mean ± SEM. A *p*-value of <0.05 was considered statistically significant. *p*-values were only indicated for significant or marginally significant results. (**b**) Representative fields of hematoxylin-and-eosin-stained liver cross-sections of each treatment group. Magnification is 10 × 20. The steatotic changes are seen as clear vacuoles occupying the hepatocytes.

To examine the effects of hgd40 at a cellular level, liver histopathological features were examined. Interestingly, treatment of *db*/*db* mice with 100 µg/mL hgd40 resulted in a marked decrease in the percentage of hepatocytes exhibiting ballooning degeneration (Figure 5a; *p* = 0.0173), a form of hepatocytic injury frequently observed in some liver conditions, especially non-alcoholic steatohepatitis [45]. In contrast, pioglitazone and hgd40 (10 µg/mL) did not exhibit any significant effects (Figure 5a). While none of the treatments had a marked effect on fatty changes in hepatocytes, 100 µg/mL of hgd40 increased the percentage of fatty cells in the liver with borderline significance (Figure 5a, *p* = 0.0746). Figure 5b contains a representative microscopic field from each treatment group in Figure 5a.

### 3.6. Treatment with GATA-3 DNAzyme Tends to Upregulate Il10 in the Liver of db/db Mice

**Figure 6:**
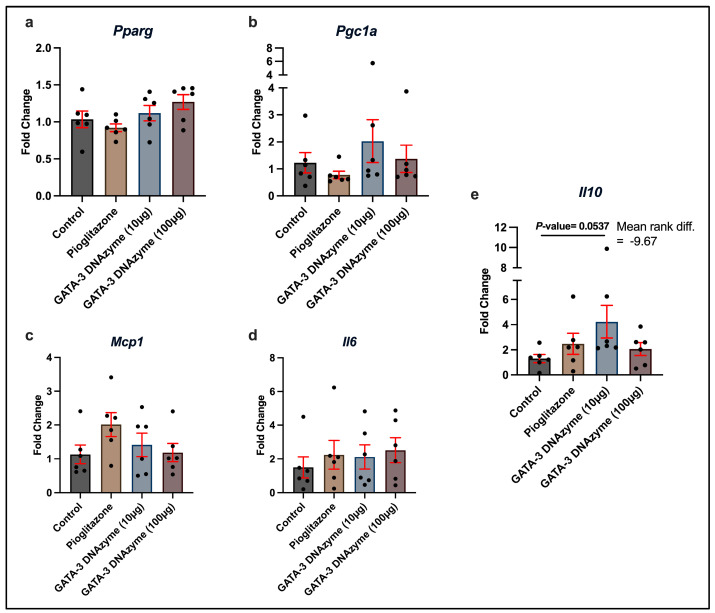
Relative mRNA expression of selected genes in the liver of *GATA-3* DNAzyme-treated *db*/*db* mice. Reverse transcriptase PCR (RT-PCR) signals for each gene were normalized to *Gapdh* as an internal control. The results are represented as fold changes relative to the control group. (**a**) peroxisome proliferator-activated receptor gamma (*Pparg*), (**b**) PPARG coactivator 1 alpha (*Ppargc1a*), (**c**) monocyte chemoattractant protein-1 (*Mcp1*) (**d**) interleukin 6 (*Il6*), and (**e**) interleukin 10 (*Il10*). Each dot represents data from a single mouse (*n* = 6/group). Statistical analyses of (**a**,**c**) were performed using one-way ANOVA, followed by Dunnett’s multiple comparisons test after ensuring normality and homogeneity of variance. Statistical analyses of (**b**,**d**,**e**) were performed using Kruskal–Wallis’s test, followed by Dunn’s multiple comparisons test due to violation of normality. All experimental groups were compared to the control group. Results are expressed as mean ± SEM. A *p*-value of <0.05 was considered statistically significant. *p*-values were only indicated for significant or marginally significant results.

To investigate the downstream effects of hgd40 on the liver of *db*/*db* mice at the molecular level, mRNA expression of a selected group of genes related to IR, adipogenicity, and inflammation was assessed. The relative mRNA expression of *Pparg*, *Pgc1a*, *Il6* and *Mcp1* did not change significantly upon pioglitazone or hgd40 treatment (Figure 6a–d). On the other hand, treating *db*/*db* mice with hdg40 at 10 µg/mL led to a borderline significant (*p* = 0.053) increase in the relative mRNA expression of *Il10* in comparison to the control group (Figure 6e).

## 4. Treatment with GATA-3 DNAzyme Significantly Increases Serum IL-10 in db/db Mice

**Figure 7:**
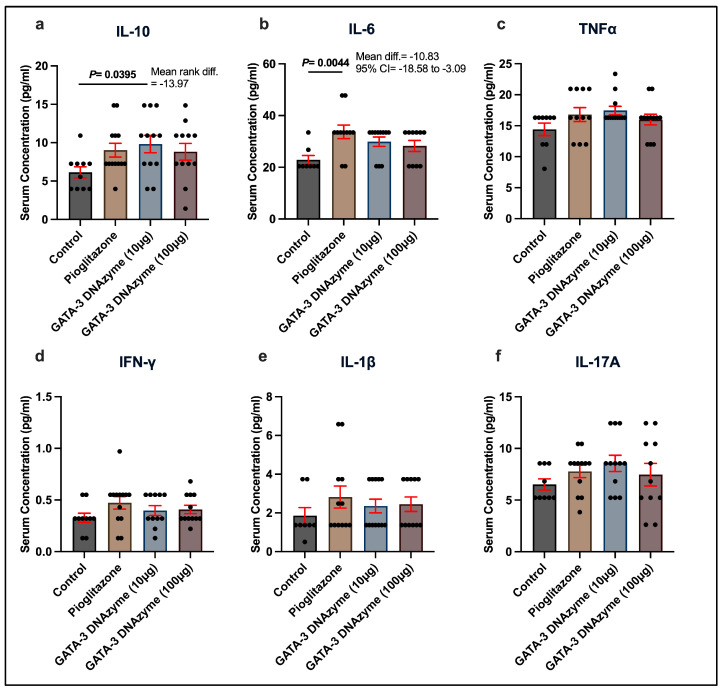
Serum concentrations of inflammation-related cytokines in *db*/*db* mice treated with GATA-3 DNAzyme. Serum concentrations of the indicated cytokines were measured at the end of the treatment period. (**a**) Interleukin-10 (IL-10), (**b**) Interleukin-6 (IL-6), (**c**) Tumor necrosis factor alpha (TNF-α), (**d**) Interferon gamma (IFN-γ), (**e**) Interleukin-1 beta (IL-1β), (**f**) Interleukin-17A (IL-17A). Each dot represents data from a single mouse (at least *n* = 8/group). Statistical analyses of (**a**,**e**) were performed using Kruskal–Wallis’s test, followed by Dunn’s multiple comparisons test due to violation of normality. Statistical analyses of (**b**–**d**,**f**) were performed using one-way ANOVA followed by Dunnett’s multiple comparisons test after ensuring normality and homogeneity of variance. All experimental groups were compared to the control group. Results are expressed as mean ± SEM. A *p*-value of <0.05 was considered statistically significant. *p*-values were only indicated for significant or marginally significant results.

To check whether GATA-3 suppression affects systemic inflammation, different inflammation-related cytokines were examined in the serum of *db*/*db* mice. Aligned with the liver-specific observations, mice treated with 10 µg/mL hgd40 had significantly higher serum levels of IL-10 compared to the control mice (Figure 7a). On the other hand, pioglitazone caused a significant elevation in the serum levels of IL-6 (Figure 7b). None of the treatments had a significant effect on any of the other tested cytokines (Figure 7c–f).

## 5. Discussion

This study suggests a possible role of GATA-3 suppression in modulating inflammation and hepatocyte injury, as demonstrated by the increased IL-10 expression and decreased ballooning degeneration in the liver. Although hgd40-mediated induction of IL-10 was previously observed in 3T3L-1 preadipocytes [28] and Wistar rat models [23], our study demonstrates that this effect extends to *db*/*db* mice. Additionally, for the Wistar rat models, hgd40 was administered through aerosol inhalation and the increase in IL-10 was demonstrated in the bronchoalveolar lavage fluid [23]. Conversely, this study shows that despite the local administration of hgd40 by subcutaneous injections, IL-10 was systemically increased in the serum and liver of *db*/*db* mice.

IL-10 is an anti-inflammatory cytokine with insulin-sensitizing properties [46,47] and several protective mechanisms against liver injury [46]. Miller et al. showed that deletion of IL-10 in mice protects them against high-fat diet-induced liver steatosis, an observation which might seem counterintuitive. However, their study revealed that, in fact, an inflammatory response is required for this protection mechanism. Specifically, the deletion of IL-10 results in IL-6/STAT3 mediated inflammatory response in the liver, decreasing steatosis. Whereas other steatosis-promoting inflammatory cytokines, such as TNF-α and IFN-γ, are also increased in mice lacking IL-10, their effect is masked by IL-6/STAT3 anti-lipogenic properties [48]. Therefore, the upregulation of IL-10 following GATA-3 suppression may lead to variable outcomes.

**Figure 8:**
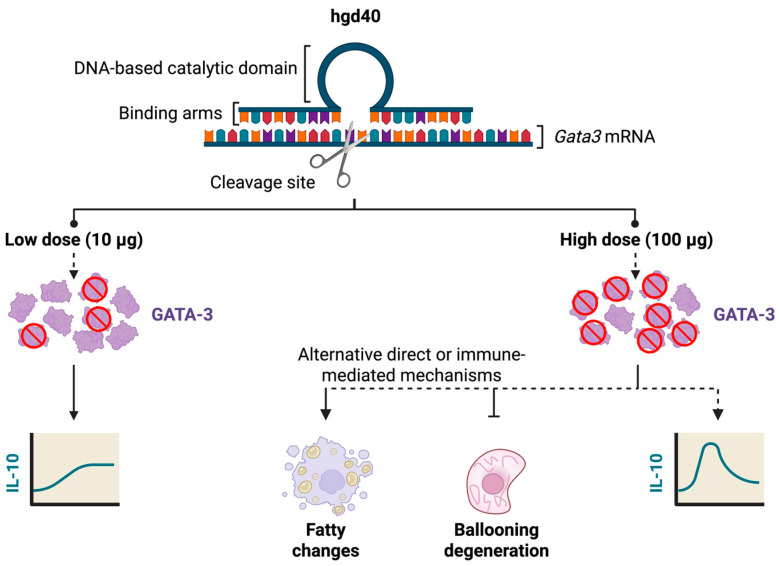
Possible mechanisms of hgd40 in *db*/*db* mice. Solid lines indicate procedures that were performed or results that were obtained in this study. Dotted lines indicate biological processes that are hypothesized to occur in response to the indicated treatments. ┴ and crossed circles indicate inhibition.

While a significant effect of hgd40 on IL-10 was observed with the low dose, the effect of the high dose did not reach statistical significance, but it followed a similar trend, especially in the serum. Despite that, only the high dose of hgd40 resulted in histopathological changes in the liver. Interestingly, it induced a steatogenic phenotype which would be expected with IL-10 upregulation. A plausible explanation could be that at a low dose of hgd40, the increase in IL-10 is mild or late and thus, was insufficient to induce histopathological changes in our experimental timeframe. However, with a high dose, IL-10 might have spiked early in the treatment period, resulting in acute effects, and then returned to near-normal levels at the time of sacrifice. More studies that investigate IL-10 dynamics and its downstream effects are required to support this hypothesis. Alternatively, hgd40’s high dose might have activated alternative mechanisms that phenocopy IL-10. Nevertheless, hgd40-mediated lipid accumulation in the liver is consistent with the literature reporting GATA-3 downregulation in fatty liver conditions [33,34]. Figure 8 summarizes these alternative hypotheses.

Even though hgd40’s high dose is steatogenic, it decreased ballooning degeneration, a histopathological feature that is characterized by multiple subcellular changes that indicate hepatocyte injury. This feature is associated with different forms of liver injury, a higher risk of liver-related complications and mortality as well as obesity, IR and necroinflammation [35,45]. A ballooning phenotype can be initiated and exacerbated by IL-1β and TNFα [36], two cytokines that are downregulated by IL-10 [37]. However, there is no direct evidence in the literature for an inhibitory effect of IL-10 on ballooning degeneration. Hence, although a transient spike in IL-10 could be one possible explanation for the decreased percentage of ballooning hepatocytes, it is very likely that other mechanisms play a major role in this. For example, hgd40 may confer direct cellular protection in the liver, perhaps by modifying local immune responses, fibrogenic signaling, or cellular stress responses, especially considering the involvement of GATA-3 in liver fibrosis as demonstrated by Xie et al. [27]. Collectively, GATA-3 suppression can lead to seemingly contradictory outcomes in terms of liver health, but in any case, these outcomes may reflect a general anti-inflammatory state. However, they may also involve different regulatory pathways, such as those related to cytoskeletal stabilization, metabolic stress or death signaling.

Site-specific assessment of *GATA-3* mRNA expression did not show a region-specific downregulation of GATA-3. Since we have previously validated the ability of hgd40 to suppress GATA-3 in 3T3L-1 mouse preadipocytes [28], the lack of difference in the current model could be explained by the dynamics of hgd40 and the turnover rate of *GATA-3* mRNA. Since the final dose of hgd40 was administered three days prior to mice sacrifice and tissue collection, it is likely that the transient nature of hgd40’s suppressive effect on *GATA-3* mRNA expression contributed to the absence of significant changes at the time of analysis. Previous in vitro and in vivo studies [20,49,50] have demonstrated that hgd40 exhibits rapid cellular uptake followed by a relatively short-lived suppression of *GATA-3* mRNA levels, with expression returning to baseline within a few days. Consequently, the timing of sample collection may have missed the critical window during which *GATA-3* mRNA suppression was most pronounced. Despite this, the observed long-lasting physiological and histopathological effects, particularly in the liver and serum, suggest that transient downregulation of GATA-3 can trigger sustained downstream biological responses. This underscores the need for future studies to closely monitor the dynamics of hgd40 uptake, *GATA-3* expression over time, and the temporal relationship between molecular suppression and functional outcomes to fully understand the therapeutic impact of GATA-3 inhibition.

Interestingly, 10 µg/mL hgd40 accelerated weight gain in the first week of the experiment despite the lack of difference in food intake and initial total body weight compared to the control. These results indicate that GATA-3 suppression with low-dose hgd40 treatment may have a transient acute effect on food absorption, metabolism, or activity. Our previous observation that the suppression of GATA-3 promotes adipogenesis, improves insulin sensitivity, and modulates inflammatory pathways makes this explanation plausible as these processes can change how the body absorbs, stores or metabolizes nutrients, independent of food intake [28,31].

Despite the prominent effect of GATA-3 suppression on the liver, neither dose of hgd40 changed fat distribution in the examined depots or the weight of any of the tested organs. This could potentially be attributed to the short intervention period although as demonstrated in our previous study, this period was sufficient to affect fat-remodeling in lean BALB/c mice [31]. Another possible reason is the choice of mouse model. Transgenic *db*/*db* mice, which lack the leptin receptor, are highly prone to obesity, becoming obese as early as five weeks of age and reaching peak weight between 10 and 16 weeks [51,52,53,54,55]. Our experiments were conducted on *db*/*db* mice aged 12 to 16 weeks, a period when fat depots in these animals may already be saturated or nearly saturated. The observed intra-group variation in mice weight gain further supports this assumption. Therefore, our results indicate that subcutaneous injection of 10 µg/mL or 100 µg/mL hgd40 cannot reverse the predetermined fat distribution in obese *db*/*db* mice. Yet, to investigate whether GATA-3 suppression can affect fat distribution during the accumulation phase of fat depots, a diet-induced obesity model can be used as obesity can be induced by a high-fat diet during the experimental period. This allows for GATA-3 DNAzyme treatment in parallel with the accumulation of body fat, capturing a critical period of adipogenesis.

Conversely, treating age-matched *db*/*db* mice with the positive control, pioglitazone, tended to increase the weights of both the omental and inguinal adipose tissues, suggesting its ability to redistribute fat depots. This comes in line with the previously described changes in fat depots of *fa*/*fa* rats and total fat mass of *db*/*db* mice treated with pioglitazone [45,56]. By activating PPARγ, pioglitazone can promote the formation of new, small, metabolically active adipocytes, leading to increase in fat mass and sequestering lipids away from more harmful stores near insulin-responsive organs, such as muscles. As a result, pioglitazone improves insulin sensitivity and overall metabolic health, despite increasing fat mass [56]. However, while pioglitazone increased the weight of BAT in *fa*/*fa* rats [56] and diet-induced obese mice [57], it decreased the weight of BAT in our study model. A previous study has reported elevated *UCP-1* (browning-associated marker) expression levels upon pioglitazone treatment of *db*/*db* mice and humans [45], but the specific effect on the weight of BAT was not mentioned. Therefore, further functional investigation might aid in the interpretation of the results observed in our study.

This study had some limitations. Firstly, the mice were group- rather than individually caged and therefore, the exact food intake of each mouse could not be determined. While at least 2–3 cages were used per group, allowing for intergroup comparison, this distribution can still mask individual responses which may skew the results. Secondly, our study is limited by the inability to capture GATA-3 suppression at the time of sacrifice. Since all mouse models dedicated to the study had already been sacrificed, it was not possible to reassess *GATA-3* expression at timepoints that are closer to the last administered dose. Accordingly, results should be treated with caution and future studies should closely monitor hgd40 uptake and *GATA-3* expression dynamics in targeted tissues at earlier timepoints. Moreover, the effect of hgd40 on *GATA-3* expression could have been suboptimal as *db*/*db* mice already lack leptin receptor, and leptin is a known promoter of GATA-3. However, Guan et al. have shown that leptin receptor-deficient obese mice can still express GATA-3 to certain level [58], suggesting that GATA-3 might still be expressed and functioning in *db*/*db* mice. Another limitation is the lack of metabolic marker measurements, such as glucose and insulin levels, which were not assessed. Hence, further experiments are needed to link the observed effects with insulin resistance and T2D.

## 6. Conclusions

This study examined the effects of GATA-3 suppression using a DNAzyme (hgd40) in obese *db*/*db* mice. While no significant changes in body fat distribution or organ weights were observed, notable effects on liver histopathology and cytokine expression occurred. High-dose hgd40 reduced hepatocyte ballooning degeneration, but caused a borderline increase in fatty changes, suggesting a dual role of GATA-3 in obesity-related liver injury. Consistent with this, low-dose hgd40 increased the expression of IL-10, suggesting anti-inflammatory activity that can influence steatosis. These findings highlight the possible utility of GATA-3 suppression to modulate liver complications associated with obesity. Yet, differences from studies in lean mice and the possibility of GATA-3’s dual role underscore the complexity of obesity models and the need for more detailed studies. Future research will explore alternative models and extended treatment durations to clarify its therapeutic potential.

## 7. Patents

The authors declare a patent involved in the reported work (US Patent App. US11976282B2).

## Figures and Tables

**Table 1:** The list of forward and reverse primers used for gene expression analysis.

Gene	Primers Sequences (5′ to 3′)
*Gapdh*	*f*: AGGTCGGTGTGAACGGATTTG
	*r*: TGTAGACCATGTAGTTGAGGTCA
*GATA-3*	*f*: GAACCGGCCCCTTATCAAG
	*r*: ACAGTTCGCGCAGGATGTC
*Pparγ*	*f*: GGCTTCCACTATGGAGTTCA
	*r*: GATCCGGCAGTTAAGATCAC
*Pgc-1α*	*f*: TGCAGCCAAGACTCTGTATG
	*r*: ATTGGTCGCTACACCACTTC
*Mcp1*	*f*: GCTACAAGAGGATCACCAGCAG
	*r*: GTCTGGACCCATTCCTTCTTGG
*Il6*	*f*: TAGTCCTTCCTACCCCAATTTCC
	*r*: TTGGTCCTTAGCCACTCCTTC
*Il10*	*f*: GCTCTTACTGACTGGCATGAG
	*r*: CGCAGCTCTAGGAGCATGTG

## Data Availability

The datasets used and/or analyzed during the current study are available from the corresponding author on reasonable request.
